# Synthesis and radiolabelling of PSMA-targeted derivatives containing GYK/MVK cleavable linkers

**DOI:** 10.1098/rsos.220950

**Published:** 2023-03-08

**Authors:** Erika Murce, Erik de Blois, Sophie van den Berg, Marion de Jong, Yann Seimbille

**Affiliations:** ^1^ Department of Radiology and Nuclear Medicine, University Medical Center Rotterdam, Erasmus MC, Rotterdam, The Netherlands; ^2^ Erasmus MC Cancer Institute, Rotterdam, The Netherlands; ^3^ TRIUMF, Life Sciences Division, Vancouver, Canada

**Keywords:** prostate cancer, targeted radionuclide therapy, prostate-specific membrane antigen, albumin binder, cleavable linkers, solid phase peptide synthesis

## Abstract

Targeted radionuclide therapy (TRT) is a promising strategy to treat different types of cancer. TRT relies on a targeting vector used to deliver a therapeutic radionuclide specifically to the tumour site. Several low molecular weight ligands targeting the prostate-specific membrane antigen (PSMA) have been synthesized, but their pharmacokinetic properties still need to be optimized. Hereby, we describe the synthesis of new conjugates, featuring the cleavable linkers Gly-Tyr-Lys (GYK) and Met-Val-Lys (MVK), to reduce the dose delivered to the kidneys. Compounds were synthesized by solid-phase peptide synthesis (SPPS) and obtained in greater than 95% chemical purity. Radiolabelling was performed with both In-111 and Lu-177 to validate potential use of the compounds as both imaging and therapeutic agents. Radiochemical purity greater than 80% was obtained for both nuclides, but significant radiolysis was observed for the methionine-containing analogue. The results obtained thus far with the GYK-PSMA conjugate could warrant further biological investigations.

## 1. Introduction

Prostate cancer is the second most frequent cancer in men worldwide, with approximately 1 400 000 new cases and 375 000 deaths reported in 2020 [1]. Prognosis is particularly poor in patients with metastatic castration-resistant prostate cancer, leading to high clinical demand for effective treatment. Targeted radionuclide therapy (TRT) is emerging as a promising therapeutic strategy for different types of cancers [2]. In 2018, Lutathera ([^177^Lu]Lu-DOTA-TATE) became the first radiopharmaceutical approved by the FDA and EMA for the treatment of neuroendocrine tumours affecting the digestive tract [3]. Prior to approval, many patients had already benefited from this treatment in a clinical setting. TRT targets receptors overexpressed at the surface of cancer cells using peptide-derived vectors. Therapeutic radionuclides, such as the β^−^-emitter Lu-177 or the α-emitter Ac-225, are conjugated to these vectors via a chelator to deliver radiation to tumour cells, ultimately leading to cell death. They can also be radiolabelled with diagnostic radionuclides, such as Ga-68 or In-111, giving rise to theranostic drugs (therapy + diagnostic) [4].

**Figure 1.**
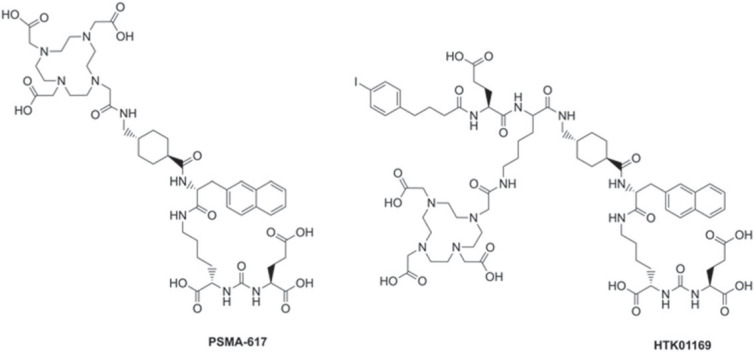
Structures of PSMA-617 and HTK01169. To form HTK01169, the linker of PSMA-617 was modified by including a lysine, a glutamic acid and the albumin binder 4-(p-iodophenyl)butyric acid. The DOTA chelator was attached to the side chain of the lysine residue.

The prostate-specific membrane antigen (PSMA) is overexpressed in prostate cancer cells, and its expression increases with disease progression [5]. PSMA is therefore an attractive target for TRT of prostate cancer. Currently, several compounds targeting this protein are being investigated for imaging and treatment of prostate cancer, with PSMA-617 advancing as a lead candidate (figure 1) [6,7]. Results of a prospective phase II clinical trial, Lu-PSMA (Australian New Zealand Clinical Trials Registry, no. 12615000912583) showed high response rates, low toxic effects and pain reduction in patients treated with [^177^Lu]Lu-PSMA-617 [8]. A prospective multi-centre phase III clinical study (Vision, ClinicalTrials.gov Identifier: NCT03511664) comparing [^177^Lu]Lu-PSMA-617 plus standard of care with current standard of care showed an increase in overall survival (median 15.3 versus 11.3 months) and imaging-based progression free survival (median 8.7 versus 3.4 months) in the [^177^Lu]Lu-PSMA-617 plus standard of care group. However, incidence of adverse effects (grade 3 or 4) in patients treated with [^177^Lu]Lu-PSMA-617 was higher than in patients treated only with standard of care (52.7% versus 38.0%). Moreover, despite encouraging response rates of over 50% [8], approximately 30% of the patients treated with [^177^Lu]Lu-PSMA-617 do not respond significantly to the treatment. Studies with [^225^Ac]Ac-PSMA-617 are currently gaining attention, as the α-emitter is expected to be more lethal to cancer cells [9]. However, the injected dose of these radiopharmaceuticals, established through dosimetric studies, is still limited by off-site accumulation. A dose of 7.4 GBq per cycle of treatment for [^177^Lu]Lu-PSMA-617 is often employed [10], with the limiting organs being the kidneys and the salivary glands [11], despite the current lack of a consensus on the ideal dose per cycle [12].

To overcome these issues, researchers are focusing on the improvement of the pharmacokinetics of these agents by conjugating an albumin-binding moiety to the PSMA-targeting vector to increase the blood circulation and tumour uptake of the radiopharmaceutical [13–16]. The injected dose can then be decreased while retaining the same uptake. Nevertheless, the shortcoming of this strategy is that it also increases the uptake in the kidneys, among other healthy organs, leading to higher radiotoxicity. To address this limitation, we employed a strategy first described by Arano *et al*. [17] based on a cleavable linker. The linker is cleaved by renal brush border enzymes, limiting the residence time of the radiopharmaceutical in the kidneys.

In this study, we synthesized PSMA-targeted radiopharmaceuticals that combined an albumin binder and a cleavable linker to optimize their therapeutic efficacy. We selected the albumin-binder containing PSMA-617 derivative HTK01169 (figure 1), reported by Kuo *et al*., as a reference [13]. [^177^Lu]Lu-HTK01169 delivers an eightfold higher dose to the tumour than [^177^Lu]Lu-PSMA-617, but also a 17-fold higher dose to the kidneys [13]. The amino acid sequences Gly-Tyr-Lys (GYK) and Met-Val-Lys (MVK) were selected as the cleavable linkers. Effective cleavage of the MVK sequence in ^67/68^Ga-labelled antibody fragments has been demonstrated by Arano *et al.* [17], and recent studies with ^67/68^Ga-labelled peptides have shown that this strategy can also be applied to small molecules [18–20]. The GYK sequence has recently been reported to be cleaved by renal brush border enzymes, when attached to Cy5 [21]. We selected DOTA (2,2′,2′′,2′′′-(1,4,7,10-tetraazacyclododecane-1,4,7,10-tetrayl)tetraacetic acid) as a chelator, to allow efficient radiolabelling with various radiometals. The DOTA chelator was attached to the peptides via an amide bond, similarly to other conjugates reported in the literature [20]. All compounds were radiolabelled with both indium-111 and lutetium-177 in order to verify their suitability as imaging (^111^In) and therapeutic (^177^Lu) agents.

## 2. Material and methods

### 2.1. General methods

All chemicals and solvents were obtained from commercial suppliers and were used without further purification, unless specified. Lutetium-177 (LuMark Lutetium-177 chloride) was purchased from IDB Holland (Baarle-Nassau, The Netherlands) and indium-111 (Indium (In-111) chloride) was purchased from Curium Netherlands BV (Petten, The Netherlands). HTK01169 and its derivatives were synthesized by solid-phase peptide synthesis (SPPS), with the exception of certain steps performed in liquid phase, as specified below. Reactions were monitored by thin-layer chromatography (TLC) using silica gel 60 F254 plates (Merck; Darmstadt, Germany) and visualized under ultraviolet light or by staining with 10% phosphomolybdic acid in ethanol. Solid-phase reactions were monitored by Kaiser test. Liquid chromatography–mass spectrometry (LC-MS) analyses were performed on an Agilent 1260 Infinity II LC/MSD XT system (Amstelveen, The Netherlands) controlled by ChemStation software. Nuclear magnetic resonance (NMR) spectra were recorded in CDCl_3_ and CD_3_OD on a Bruker Ascend 600 MHz system (Leiderdorp, The Netherlands) at ambient temperature. Chemical shifts are given as *δ* values in ppm and coupling constants J are given in Hz. The splitting patterns are reported as s (singlet), d (doublet), t (triplet), m (multiplet), dd (doublet of doublets) and br (broad signal). Reverse-phase high-performance liquid chromatography (RP-HPLC) was performed with a Waters Alliance e2695 system (Etten-Leur, The Netherlands) equipped with a 2998 diode array (PDA) detector, a NaI(Tl) Scionix crystal (Bunnik, The Netherlands) connected to a Canberra Osprey multichannel analyzer and signal amplifier (Zellik, Belgium), and Empower 3 software. Radioactivity was measured using a PerkinElmer Wizard 2 γ-counter (Groningen, The Netherlands). Instant thin-layer iTLC-SG chromatography plates (Agilent; Folsom, CA, USA) were analysed by radio-chromatography with a bSCAN scanner (Brightspec; Antwerp, Belgium). Radiochemical yield (RCY), expressed as a percentage (%), is determined by iTLC and corresponds to the ratio of the peak area of the radiolabelled product and the peak area of all radiochemical species. Radiochemical purity (RCP), expressed as a percentage (%), corresponds to the ratio between the peak area of the radiolabelled product and the peak area of all radiochemical species integrated via radio-HPLC.

### 2.2. HPLC conditions

The reversed phase semi-preparative Luna C18 (250 × 10 mm, 5 µm), semi-preparative Jupiter Proteo C18 (250 × 10 mm, 10 µm) and analytical Gemini C18 (250 × 4.6 mm, 5 µm) columns from Phenomenex (Torrance, CA, USA) were used in this study. The mobile phase consisted of solvent A (0.1% trifluoroacetic acid (TFA) in H_2_O) and solvent B (0.1% TFA in acetonitrile (ACN)). Analytical runs were performed at a flow rate of 1 ml min^−1^, while purifications were performed at a flow rate of 3 ml min^−1^. For analyses, the following gradient of solvents A and B was applied: 0–3 min, 5% B; 3–23 min, 5–100% B; 23–27 min, 100% B. For purification, methods 1–3 were used as specified. Method 1: 0–3 min, 5% B; 3–23 min, 5–100% B; 23–27 min, 100% B. Method 2: 0–23 min, 35% B; 23–28 min, 35–90% B; 28–33 min, 90% B. Method 3: 0–23 min, 45% B; 23–28 min, 45–90% B; 28–33 min, 90% B. HPLC eluates were monitored for their UV absorbance at 254 nm. For LC-MS, an Agilent Infinity Lab Poroshell 120 EC-C_18_ column (3 × 100 mm, 2.7 μm) was used. The mobile phase consisted of solvent A (0.1% formic acid (FA) in H_2_O) and solvent B (0.1% FA in ACN). The following LC gradient is used for all analyses: 0–5 min, 5–100% B; 5–8 min, 100% B. The flow rate is 0.5 ml min^−1^. UV detection was performed at 254 nm.

### 2.3. Synthetic procedure

#### 2.3.1. (S)-Di-*tert*-butyl-(((2,5-dioxopyrrolidin-1-yl)oxy)carbonyl)-L-glutamate (**3**)

H-Glu(O*t*Bu)-O*t*Bu (1.33 g, 4.50 mmol) was solubilized in 10 ml ACN. Triethylamine (Et_3_N, 0.623 ml, 4.50 mmol, 1 equiv.) and *N,N*-dissuccinimidyl carbonate (1.27 g, 4.95 mmol, 1.1 equiv.) were added and the reaction was stirred overnight. The reaction was monitored by TLC (EtOAc/hexane, 3:1; *R*_f_ = 0.7). After completion, the mixture was concentrated under vacuum, resolubilized in 15 ml EtOAc and washed with a 10% aqueous citric acid solution and brine. After drying over MgSO_4_, the product was concentrated under reduced pressure and obtained as a yellowish oil (1.40 g, 74%). ^1^H-NMR (600 MHz, CDCl_3_): *δ* 1.37 (s, 9H), 1.41 (s, 9H), 1.88–1.96, (m, 1H), 2.04–2.10 (m, 1H), 2.27 (t, *J* = 7.9 Hz, 2H), 2.74 (s, 4H), 4.14 (q, *J* = 7.2 Hz, 1H), 6.31 (d, *J* = 7.8 Hz, 1H). ESI-MS *m/z* calc'd for C_18_H_29_N_2_O_9_ 400.18; found 422.30 [M + Na]^+^.

#### 2.3.2. Resin-bound lysine-urea-glutamate moiety (**5**, KuE)

To 2-chlorotrityl chloride resin (0.5 g, 1.5 mmol g^−1^ loading capacity, 1 equiv.) swollen in DCM (10 ml) for 30 min was added a solution of Fmoc-Lys(Alloc)-OH (1.02 g, 2.25 mmol, 3 equiv.) in DCM (10 ml) and *N,N*-diisopropylethylamine (DIPEA, 0.983 ml, 5.62 mmol, 7.5 equiv.). The resin was agitated overnight. After filtration and washing with DCM, the resin was capped for 45 min with 10 ml of DCM/MeOH/DIPEA (v/v/v, 80 : 15 : 5). Subsequently, the Fmoc protecting group was deprotected with 10 ml of a 20% solution of 4-methyl piperidine in DMF for 15 min. Upon reaction completion, a solution of **3** (1.40 g, 3.3 mmol, 4.4 equiv.) in DCM (10 ml) and Et_3_N (0.685 ml, 4.95 mmol, 6.6 equiv.) was added to the resin. The mixture was stirred overnight followed by the Alloc deprotection with a solution of Pd(PPh_3_)_4_ (0.26 g, 0.22 mmol, 0.3 equiv.) and morpholine (1.96 ml, 22.5 mmol, 30 equiv.) in DCM (10 ml). The reaction mixture was stirred for 3 h. Then, the resin was washed once with 1% DIPEA in DMF, followed by five times with 10% sodium diethylthiocarbamate in DMF to remove the palladium. Deprotection was monitored by LC-MS. A sample of this compound was cleaved from solid phase for LC-MS and NMR characterization using a TFA/TIPS (triisopropylsilane)/H_2_O cocktail. ^1^H-NMR (600 MHz, CD_3_OD): *δ* 1.46 (s, 9H), 1.49 (s, 9H), 1.39–1.54 (m, 2H), 1.65–1.74 (m, 3H), 1.79–1.87 (m, 2H), 2.02–2.08 (m, 1H), 2.28–2.38 (m, 2H), 2.93 (t, *J* = 7.5 Hz, 2H), 4.14–4.16 (m, 1H), 4.18–4.21 (m, 1H). ESI-MS *m/z*: calc'd for C_20_H_38_N_3_O_7_ 431.26; found 432.20 [M + H]^+^.

#### 2.3.3. EuK(2-Nal-Trx-Lys-Glu-4-*p*-IPBA) (**6**)

Synthesis of **6** was performed by elongation of **5** (0.75 mmol, 1 equiv.) according to Kuo *et al*. [13]. Conjugation of each Fmoc-protected amino acid was achieved by adding a solution of the amino acid (3 mmol, 4 equiv.) in DMF (10 ml) with HBTU (*O*-(benzotriazol-1-yl)-*N*,*N*,*N′*,*N′*-tetramethyluronium hexafluorophosphate; 1.15 g, 2.94 mmol, 3.9 equiv.) and DIPEA (1.04 ml, 6 mmol, 8 equiv.). The resin was agitated for 2 h. Fmoc-groups were deprotected as previously described with a 20% solution of 4-methyl piperidine in DMF. Incomplete steps were repeated. Fmoc-2-Nal-OH, Fmoc-tranexamic acid, Fmoc-Lys(ivDde)-OH, Fmoc-Glu(tBu)-OH and 4-(*p*-iodophenyl)butyric acid were coupled using this protocol. Deprotection of the ivDde (1-(4,4-dimethyl-2,6-dioxocyclohex-1-ylidene)-3-methylbutyl) group with a 5% hydrazine solution in DMF provided the key intermediate **6**, which was directly used without further purification.

#### 2.3.4. HTK01169

DOTA-tris(*t*Bu) ester (114 mg, 0.20 mmol, 2 equiv.), HBTU (74 mg, 0.20 mmol, 2 equiv.) and DIPEA (70 μl, 0.40 mmol, 4 equiv.) in DMF (5 ml) were added to the peptidyl-resin **6** (0.10 mmol, 1 equiv.) and stirred overnight. The reaction was monitored by LC-MS. The product was cleaved from the solid support by treatment of the resin with a solution of TFA/TIPS/H_2_O (5 ml, v/v/v = 95 : 2.5 : 2.5) for 2 h. The resin was washed twice with 5 ml of the cleavage cocktail. The solution was concentrated with a gentle air flow, and the product was precipitated by addition of ice-cold diethyl ether. The mixture was centrifuged for 20 min (3000*g*) and the ether phase was removed. This process was repeated twice. After drying, the crude peptide was purified by semi-preparative HPLC (Jupiter Proteo C18 column, Method 1, *t*_R_ = 18.5 min) to give HTK01169 as a white solid (42 mg, 27% yield, purity greater than 95%). ESI-MS *m/z*: calc'd for C_70_H_100_IN_12_O_21_ 1570.60; found 1571.30 [M + H]^+^.

#### 2.3.5. EuK(2-Nal-Trx-Lys[Gly-Tyr-Lys(DOTA)]-Glu-4-*p*-IPBA) (**7**)

Fmoc deprotection and coupling of Fmoc-Gly-OH, Fmoc-Tyr(*t*Bu)-OH and ivDde-Lys(Fmoc)-OH onto the peptidyl-resin **6** (0.10 mmol) were performed as reported above for **6**. ivDde deprotection, DOTA attachment and cleavage of the peptide from the resin were carried out as described for HTK01169. The precipitate was treated for an additional 2 h with TFA/TIPS/H_2_O (v/v/v = 95 : 2.5 : 2.5) to remove all *tert*-butyl protecting groups. Purification of **7** was achieved by semi-preparative HPLC (Luna C18 column, Method 2, *t*_R_ = 13.80 min) to yield compound **7** (19 mg, 10% yield, purity greater than 95%) as a white solid. ESI-MS *m/z*: calc'd for C_87_H_123_IN_16_O_25_ 1918.79; found 1920.40 [M + H]^+^.

#### 2.3.6. EuK(2-Nal-Trx-Lys[Met-Val-Lys(DOTA)]-Glu-4-*p*-IPBA) (**8**)

**Scheme 1.**
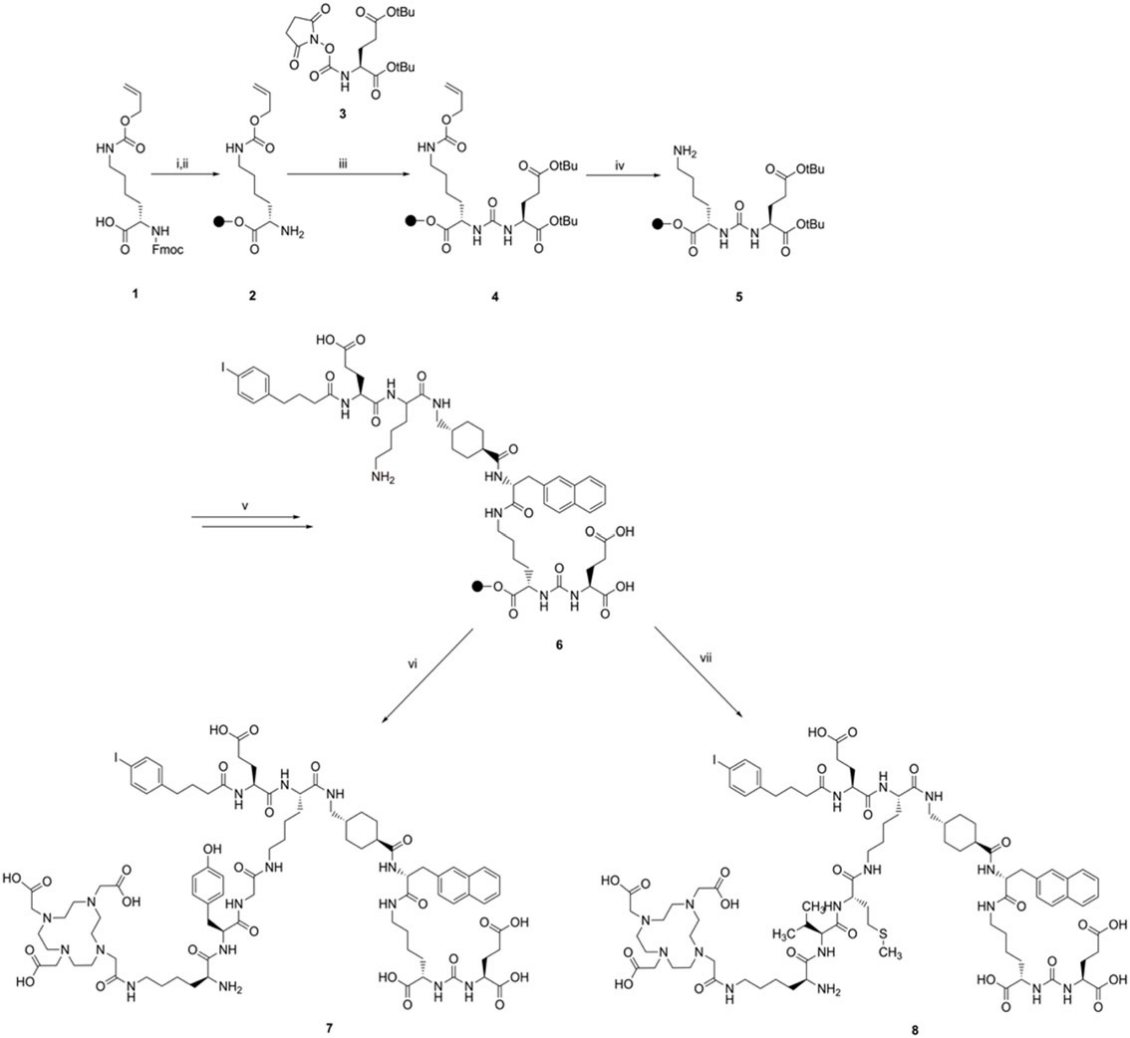
Synthetic route for compounds **7** and **8**. The resin used for SPPS is indicated as a black sphere in the scheme. Reagents and conditions: (i) 2-chlorotrityl chloride resin, DCM, overnight, r.t.; (ii) 20% 4-methyl piperidine in DMF, 15 min, r.t.; (iii) **3**, Et_3_N, DCM, overnight, r.t.; (iv) Pd(PPh_3_)_4_, morpholine, DCM, 3 h, r.t.; (v) elongation of the peptide chain with Fmoc-2-Nal-OH, Fmoc-tranexamic acid, Fmoc-Lys(ivDde)-OH, Fmoc-Glu(tBu)-OH and 4-(p-iodophenyl)butyric acid; (vi) elongation of the peptide chain with Fmoc-Gly-OH, Fmoc-Tyr(tBu)-OH, ivDde-Lys(Fmoc)-OH and DOTA-tris(tBu) ester; (vii) elongation of the peptide chain with Fmoc-L-Met-OH, Fmoc-Val-OH, ivDde-Lys(Fmoc)-OH and DOTA-tris(tBu) ester.

Synthesis of **8** was performed according to the method described above for **7**. Elongation of **6** with Fmoc-Met-OH, Fmoc-Val-OH and ivDde-Lys(Fmoc)-OH provided **8**. After semi-preparative HPLC purification (Luna C18 column, Method 3, *t*_R_ = 14.00 min), **8** (12 mg, 5.8% yield, purity greater than 95%) was isolated as a white solid. ESI-MS *m/z*: calc'd for C_86_H_129_IN_16_O_24_S 1928.81; found 1930.50 [M + H]^+^ (scheme 1).

### 2.4. Radiochemistry

#### 2.4.1. Labelling with [^111^In]InCl_3_

Labellings were performed in Milli-Q water (final volume: 140 μl) to which was added sodium acetate (1 μl, 2.5 M), ascorbic and gentisic acids (10 μl, 50 mM), and 1 nmol of HTK01169, **7** or **8** solubilized in H_2_O/ACN (1 : 1). Concentration of the precursors in solution was determined via titration [22,23]. [^111^In]InCl_3_ (20 MBq, 370 MBq ml^−1^) was added and the mixture was incubated at 90°C for 20 min. Quality control was performed using silica-gel-coated instant thin-layer chromatography (iTLC-SG) eluted with a solution of sodium citrate (0.1 M, pH 5.0). Diethylenetriaminepentaacetic acid (DTPA; 5 μl, 4 mM) was added to complex-free In-111 before injection onto analytical HPLC.

#### 2.4.2. Labelling with [^177^Lu]LuCl_3_

Radiolabelling with [^177^Lu]LuCl_3_ (20 MBq) was performed in analogous conditions, but with addition of L-methionine (10 μl, 50 mM) to the reaction mixture. Quality control was performed similarly to the indium-111 labelling by iTLC analysis followed by injection onto analytical HPLC after addition of DTPA.

#### 2.4.3. Radiochemical stability in phosphate buffered saline and mouse serum

Stability was determined for both the In-111 and Lu-177 labelled compounds by mixing 20 µl of radiolabelled sample (approx. 5 MBq) with 80 µl phosphate buffered saline (PBS) (0.01 M, pH 7.4), or mixing 70 µl of radiolabelled sample (approx. 20 MBq) with 330 µl of mouse serum, and heating at 37°C for up to 24 h. After incubation, 50 µl of the mouse serum solution is added to a separate vial containing acetonitrile (50 µl) and the sample is centrifuged for 20 min. The supernatant and precipitate are separated, and the activity is measured to determine the binding to serum proteins. The supernatant is then analysed by radio-HPLC. The PBS solution is loaded directly onto the radio-HPLC.

## 3. Results and discussion

The purpose of this study is to report the synthesis and radiolabelling of new PSMA derivatives containing: (i) a PSMA-targeting vector, (ii) an albumin binder, namely 4-(*p-*iodophenyl)butyric acid, (iii) an amino acid sequence which could be specifically recognized and cleaved by renal brush border (RBB) enzymes, and (iv) a DOTA chelator which can be labelled with In-111 for imaging or Lu-177 for therapy. Previous studies reported that presence of an albumin-binding moiety increases blood residence time of the radiopharmaceutical agent, and ultimately leads to a higher accumulation of the PSMA radioligand in the tumour compared with agents without an albumin binder [13,14]. A similar therapeutic effect can therefore be achieved while injecting a lower dose of activity. This could theoretically result in less side effects during radionuclide therapy, but also less exposure of the healthcare workers while handling radioactive materials. The choice of the albumin binder significantly influences blood residence time of the radiopharmaceutical. A strong binding to albumin will lead to a high retention of the radiopharmaceutical in the blood, while selection of a weaker albumin binder will result in a faster clearance from the blood pool [16,24]. The low molecular weight compound 4-(*p*-iodophenyl)butyric acid has been shown to possess good affinity to both mouse and human serum albumin [25]. Folate-receptor targeting agents [26], as well as PSMA radioligands [14,16], demonstrated improved bioavailability when conjugated to 4-(*p*-iodophenyl)butyric acid. In particular, Benešová *et al*. showed that [^177^Lu]Lu-PSMA-ALB-02 had enhanced blood circulation and higher tumour uptake compared with [^177^Lu]Lu-PSMA-617 [14]. However, uptake in the kidneys was considerably higher for [^177^Lu]Lu-PSMA-ALB-02 with approximately 60% of the injected activity per gram (IA g^−1^) at 1 h and approximately 11% IA g^−1^ at 24 h post-injection (p.i.) versus approximately 10% IA g^−1^ at 1 h and less than 1% IA g^−1^ at 24 h p.i. for [^177^Lu]Lu-PSMA-617. Kuo *et al*. also demonstrated that the 4-(*p*-iodophenyl)butyryl-containing PSMA ligand ([^177^Lu]Lu-HTK01169) delivered an eightfold higher radiation dose to tumours compared with [^177^Lu]Lu-PSMA-617 [13]. However, it was accompanied by a 17-fold higher radiation dose to the kidneys. The improvement of tumour uptake seen in these studies led us to select 4-(*p*-iodophenyl)butyryl as our albumin binder, despite the concomitant increased kidney uptake. Recently, other similar albumin binders have been evaluated and Kuo *et al*. reported that Ga-68 labelled derivatives of HTK01169 containing *N*-(4-(*p*-chlorophenyl)butanoyl)-Gly and *N*-(4-(*p*-methoxyphenyl)butanoyl)-Gly motifs showed lower average kidney uptake (approx. 55% IA g^−1^) with moderate blood retention and relatively faster tumour uptake (approx. 30% IA g^−1^ at 3 h p.i.) [27], illustrating the key role of albumin binders in modulating the pharmacokinetic properties of a radiopharmaceutical.

High kidney uptake of radiopharmaceuticals can usually be explained by the long residence time of radiometabolites generated after lysosomal proteolysis. This process is followed by glomerular filtration and reabsorption of the radiometabolites in renal cells [28]. By inserting a linker which is specifically cleaved by enzymes on the brush border membrane located in the lumen of the proximal renal tubule, the radiometal-chelator moiety can be rapidly excreted via the bladder while the peptide remains in the kidneys. The MVK sequence was initially conjugated to antibody fragments by Uehara *et al*., and the linker was cleaved between the methionine and valine by the neutral endopeptidase (NEP) enzyme, highly expressed in the renal brush border membrane [18]. The authors demonstrated that this linker is stable in plasma and the radioactivity levels delivered to tumours were not influenced by the addition of the linker. Recent studies by Cleeren *et al*. on the GYK and GFK linkers showed that quantitative cleavage was obtained with the Cy5 labelled model peptide, H-GGGYK(Cy5)-NH_2_, while incubation of H-GGGFK(Cy5)-NH_2_ with RBB enzymes resulted in 82% cleavage [21]. The GFK sequence had already been investigated and was efficiently cleaved when attached to antibody fragments [29,30]. These promising results encouraged us to select the MVK and GYK cleavable linkers for our compounds.

Protocols for the general synthesis of the PSMA-moiety (lysine-urea-glutamate, KuE, **5**) and HTK01169 were adapted from the literature [13,31]. Synthesis of **5** started with the attachment of Fmoc-Lys(Alloc) to the resin. After Fmoc deprotection, a solution of (S)-di-*tert*-butyl-(((2,5-dioxopyrrolidin-1-yl)oxy)carbonyl)-L-glutamate (**3**) in DCM and Et_3_N was added to the resin to yield the Alloc-protected KuE intermediate (**4**). The liquid-phase synthesis of **4** was efficiently adapted to solid phase and provided **4** in high yields (88–90%) [31]. This synthetic route employs milder reaction conditions compared with the synthesis commonly reported using triphosgene [6,13]. The Alloc-protected lysine was chosen because this protecting group can be completely removed by treatment with a solution of tetrakis(triphenylphosphine)palladium(0) and morpholine. We also attempted the synthesis with the benzyloxycarbonyl-protected lysine (Fmoc-Lys(Z)-OH), which also resulted in a satisfactory yield of 78%. However, complete deprotection of the side chain amino group of the lysine residue was longer (48 h versus 3 h) and therefore we opted for the Alloc-protected lysine. Elongation of **5** and attachment of the albumin binding group was performed by standard SPPS protocols to obtain the key intermediate **6**. Elongation of **5** was initially carried out with Fmoc-Lys(Alloc) instead of Fmoc-Lys(ivDde)-OH. However, Alloc deprotection and formation of **6** could not be identified even after 72 h of reaction. Fmoc-Lys(ivDde)-OH was then used in place of Fmoc-Lys(Alloc)-OH and complete deprotection of the ivDde group could be observed after 2 h of treatment with a solution of 5% hydrazine in DMF. HTK01169 was obtained in 27% yield after attachment of the DOTA-chelator to **6**, which is comparable to the 25% yield reported in literature [13]. Compounds **7** and **8** were obtained in 10 and 6% yield, respectively, after incorporation of the amino acids forming the cleavable linker and attachment of the chelator. The MVK analogue (**8**) was prepared by using Boc-Lys(Fmoc)-OH in place of ivDde-Lys(Fmoc)-OH. This adaptation allowed the cleavage and deprotection to be performed in a single step, as the Boc-group is acid labile. DOTA-coupling was performed with PyBOP (benzotriazol-1-yloxytripyrrolidinophosphonium hexafluorophosphate) in place of HBTU to shorten the reaction time. The coupling reaction was complete after 2 h when employing PyBOP, whereas the reaction was still incomplete after 12 h with HBTU as coupling agent. All compounds were purified by semi-preparative HPLC and their purity was above 95%. Identity of **7** and **8** was confirmed by ESI-MS. The compounds were stored as lyophilized powders, and titrated aliquots were prepared for the labellings to avoid successive freeze–thaw cycles [22].

**Figure 2.**
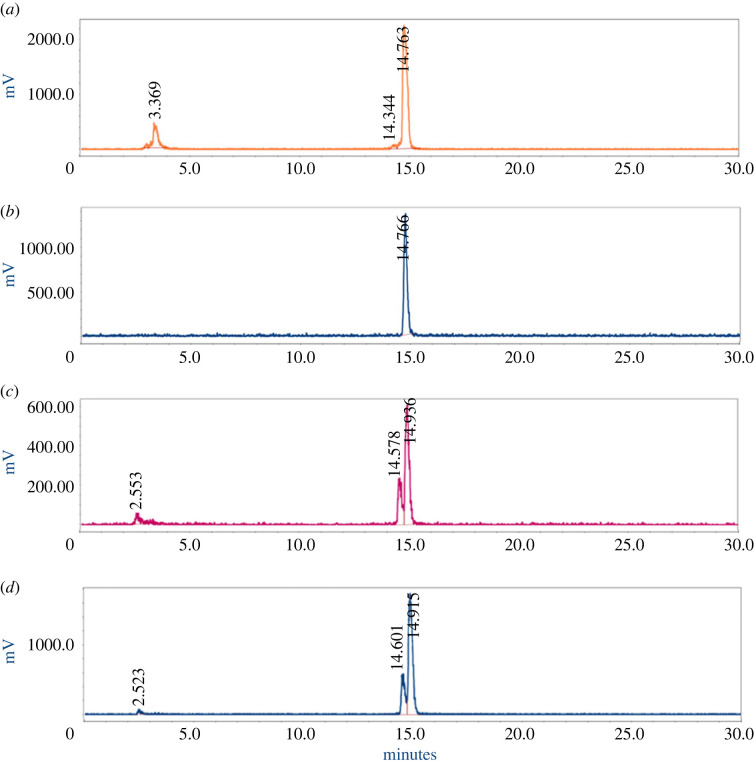
(*a*) RP-HPLC chromatogram of [^111^In]-**7**. The peak at 14.76 min corresponds to [^111^In]-**7**, while the peak at 3.37 min is [^111^In]In-DTPA. (*b*) RP-HPLC chromatogram of [^177^Lu]-**7** (*t*_R_ = 14.77 min). (*c*) RP-HPLC chromatogram of [^111^In]-**8** (*t*_R_ = 14.93 min) and its radiolysed product (*t*_R_ = 14.58 min). (*d*) RP-HPLC chromatogram of [^177^Lu]-**8** (*t*_R_ = 14.91 min) and its radiolysed product (*t*_R_ = 14.60 min). Experiments were performed in duplicate or triplicate, as specified in the text.

The metal chelator DOTA was chosen for our PSMA-targeted radioligands, as it forms stable complexes with a large variety of metals. Among others, DOTA-peptides labelled with In-111, Ga-68, Cu-64/67, Lu-177 and Y-90 have found applications in the nuclear medicine field for imaging or therapy [32]. In-111 and Lu-177 labelling were performed in sodium acetate buffer containing stabilizers. Ascorbic and gentisic acid are known to protect radiopharmaceuticals from radiolytic degradation [33,34]. Addition of methionine has also been shown to prevent radiation-induced oxidation of radiopharmaceuticals. Our group has previously reported that the combination of ascorbic and gentisic acids, as well as methionine (final concentration of 3.5 mM for each compound) is effective in maintaining a high radiochemical purity (RCP) of [^177^Lu]Lu-PSMA-617 [35,36]. Indium-111 labelling of **7** was initially performed solely with ascorbic and gentisic acids, but the RCP obtained was fairly low (60–70%). Therefore, methionine was added to the reaction mixture, which resulted in higher RCP (80.3 ± 2.2%) (figure 2*a*). These radiochemical conditions were conserved for the lutetium labelling. [^177^Lu]-**7** was obtained with a radiochemical yield and purity of greater than 95% (figure 2*b* and table 1). Radiolabelling of **8** with either ^111^In or ^177^Lu led to low radiochemical purity (57.5 ± 0.1% for [^111^In]-**8** and 70.2 ± 2.6% for [^177^Lu]-**8**), with an impurity eluting closely to the product peak on the HPLC radio-chromatograms (figure 2*c,d*). The above-mentioned values of RCP and RCY are summarized in table 1 and the iTLC chromatograms are depicted in the supplementary information (electronic supplementary material, figures S4 and S5.)

**Table 1.** Summary of the radiochemistry data^a^.

product	HPLC retention time (min)^b^	radio-HPLC retention time (min)^b^	RCY (%, In-111)	RCP (%, In-111)	RCY (%, Lu-177)	RCP (%, Lu-177)
HTK01169	14.14	15.30	93	88	99	99
**7**	13.94	14.76	90.9 ± 1.8	80.3 ± 2.2	98.9 ± 0.6	99.3 ± 0.3
**8**	14.43	14.93	83.2 ± 1.0	57.5 ± 0.1	97.1 ± 1.5	70.2 ± 2.6

^a^For all labellings, a molar activity of 20 MBq nmol^−1^ was used. The RCY and RCP of [^111^In]-**7** are calculated based on *n* = 3, and of [^177^Lu]-**7**, *n* = 3. The RCY and RCP of [^111^In]-**8** are calculated based on *n* = 2, and of [^177^Lu]-**8**, *n* = 3. Labelling of HTK-1169 was only performed once with each radiometal as a proof-of-concept.

^b^The HPLC retention times are based on injections in the analytical column (Method 1). For the radio-HPLC, in particular, the retention times shown are for the In-111 labelled compounds.

We hypothesized that this radiolabelled impurity might come from the radiation-induced oxidation of the methionine residue of the cleavable linker. Bendre *et al*. recently synthesized DOTA-AmBz-M(O)VK(HTK01166)-OH, the oxidized analogue of the PSMA-targeted derivative DOTA-AmBz-MVK(HTK01166)-OH, and performed labelling with gallium-68. They observed that the oxidized product was not cleaved by the renal brush border enzymes [20]. It confirms that oxidation of the methionine is undesirable, as it leads to loss of enzyme recognition and consequently less cleavage of the conjugate. This radiolysis-induced oxidation was observed after labelling with gallium-68 and therefore we were interested to investigate the effect of other radionuclides on the stability of the PSMA-targeted compounds containing the MVK sequence. Hernandez *et al*. [37] studied the extent of radiobleaching (decrease of the fluorescent signal due to radiolysis) of fluorescent probes conjugated to ^68^Ga, ^111^In and ^213^Bi. Interestingly, they observed that the radiolytic effect of ^68^Ga was stronger than that of ^111^In or even the alpha emitter ^213^Bi. They also investigated the use of radical scavengers during radiolabelling and found that inclusion of gentisic and ascorbic acids reduced the radiobleaching of the conjugates.

To confirm that the additional peak observed on the radio-chromatograms is effectively resulting from the oxidation of [^177^Lu]-**8**, we first tested three different concentrations of the L-methionine scavenger in the labelling solution (0, 3.5 and 7 mM). However, the effect was negligible and no significant decrease of the radiolabelled impurity could be seen (electronic supplementary material, figure S6a–c). Then, we studied the effect of adding an oxidizing agent to the precursor **8**. Chloramine-T [38,39] was added at varying concentrations (0.1, 1, 10, 100 and 1000 μM) to non-labelled **8** and analysed by LC-MS. At a concentration of 10 μM and above, a new product, identified by ESI-MS as the oxidized analogue of **8**, was formed. The formation was concentration dependent, with additional degradation of the compound observed at greater oxidizing concentrations (100–1000 μM) (electronic supplementary material, figures S7 and S8). Compound **8** was then radiolabelled with ^111^In in presence of chloramine-T (100 μM), which led to an increase of the oxidized product from 25% to 53% (electronic supplementary material, figure S9)

**Figure 3.**
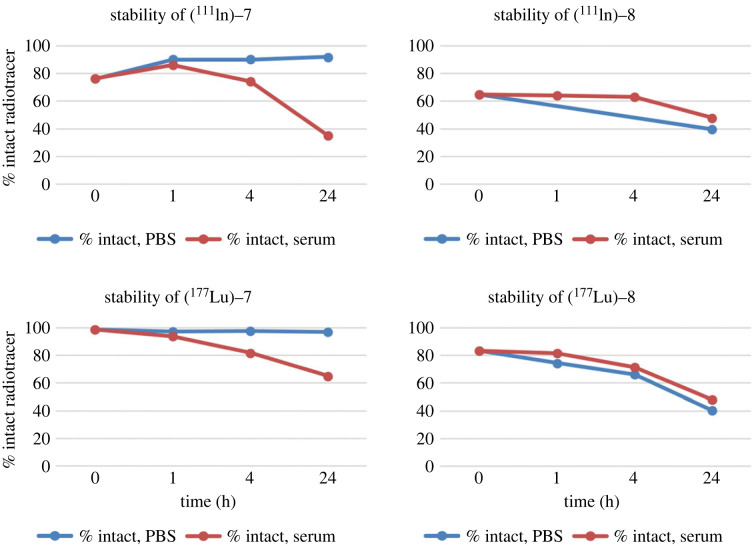
Stability over time of [^111^In]-**7**, [^111^In]-**8**, [^177^Lu]-**7** and [^177^Lu]-**8**. The percentage of intact radiotracer is plotted as a function of time (up to 24 h post-incubation), in both PBS (blue line) and mouse serum (red line).

Finally, we evaluated the stability of [^111^In]-**7** and [^111^In]-**8** in PBS buffer and mouse serum (figure 3). [^111^In]-**7** was stable in PBS solution (91%) up to 24 h. However, only 74% of the radiolabelled product remained intact after 4 h incubation in mouse serum, and 35% after 24 h (electronic supplementary material, figure S10). By contrast, the peak at 2.9 min increased proportionally to the decrease of [^111^In]-**7** (62% at 24 h). The product eluting at early retention time could correspond to the release of the radiometal from the chelator or the cleavage of a small fragment containing the chelator under these conditions. However, without additional characterization of this radiolabelled side-product, we can only speculate on the instability of these conjugates in presence of serum proteins. The same phenomenon was observed with [^111^In]-**8** in both PBS and mouse serum (electronic supplementary material, figure S11). To further investigate if the change of radiometal impacts the stability of the conjugates, stability of [^177^Lu]-**7** and [^177^Lu]-**8** was also evaluated. Following a similar trend, [^177^Lu]-**7** was stable in PBS solution (96% up to 24 h), but remained only 65% intact in mouse serum after 24 h (electronic supplementary material, figure S12). [^177^Lu]-**8** remained only 40% intact in PBS, while in serum 48% of [^177^Lu]-**8** was found at 24 h (electronic supplementary material, figure S13). For the sake of comparison, we also performed the stability of [^177^Lu]Lu-HTK01169, which was greater than 99% stable in both PBS and mouse serum up to 24 h (electronic supplementary material, figure S14). Therefore, additional studies related to the stability of these conjugates and their ability to be cleaved solely by enzymes on the brush border membrane will have to be performed before further biological evaluation can be undertaken with **7**.

Previous studies reported that both GYK and MVK linkers can result in reduction of the renal uptake of radiopharmaceuticals [19–21]. However, our data suggests that caution must be taken when employing the MVK-linker to avoid methionine oxidation, as it can lead to the presence of the M(O)VK species which is not recognized by the RBB enzymes [20]. Furthermore, it should be noted that none of these strategies, albumin binder and cleavable linker, have been clinically validated for radiopharmaceuticals, and therefore further studies are necessary to evaluate their efficacy. Indeed, one main point of concern is the increase of the dose to the bone marrow [40] for the radiopharmaceuticals containing an albumin binder. A reduced administered dose of radiopharmaceutical, as a consequence of the higher therapeutic efficacy, could possibly limit the bone marrow toxicity. Dosimetry studies will need to be performed to better support this hypothesis.

## 4. Conclusion

We have described the synthesis and radiolabelling of two PSMA-targeting conjugates, containing an amino acid sequence (Met-Val-Lys and Gly-Tyr-Lys) that can be selectively cleaved by RBB enzymes. The compounds were obtained by solid-phase peptide synthesis and purified by RP-HPLC to greater than 95% chemical purity. Compounds were characterized by ESI-MS, analytical HPLC and LC-MS. Radiolabelling of the Gly-Tyr-Lys containing compound with In-111 and Lu-177 yielded greater than 80% and 95% RCP, respectively. However, the methionine-containing compound was sensitive to radiolysis, leading to the formation of an oxidized species. Further preclinical evaluation will be performed with the Gly-Tyr-Lys containing conjugates in the near future to evaluate the binding affinity to PSMA and the cleavage of the GYK linker by the RBB enzymes.

## Data Availability

Supporting data are included in the electronic supplementary material [41].

## References

[1] Sung H, Ferlay J, Siegel RL, Laversanne M, Soerjomataram I, Jemal A, Bray F. 2021 Global cancer statistics 2020: GLOBOCAN estimates of incidence and mortality worldwide for 36 cancers in 185 countries. CA. Cancer J. Clin. **71**, 209-249. (10.3322/caac.21660) 33538338

[2] Van Essen M, Krenning EP, Kam BLR, De Jong M, Valkema R, Kwekkeboom DJ. 2009 Peptide-receptor radionuclide therapy for endocrine tumors. Nat. Rev. Endocrinol. **5**, 382-393. (10.1038/nrendo.2009.105) 19488074

[3] Strosberg J et al. 2017 Phase 3 trial of ^177^Lu-dotatate for midgut neuroendocrine tumor. N. Engl. J. Med. **376**, 125-135. (10.1056/NEJMoa1607427) 28076709 PMC5895095

[4] Yordanova A, Eppard E, Kürpig S, Bundschuh RA, Schönberger S, Gonzalez-Carmona M, Feldmann G, Ahmadzadehfar H, Essler M. 2017 Theranostics in nuclear medicine practice. Onco. Targets Ther. **10**, 4821-4828. (10.2147/OTT.S140671) 29042793 PMC5633297

[5] Schülke N et al. 2003 The homodimer of prostate-specific membrane antigen is a functional target for cancer therapy. Proc. Natl Acad. Sci. USA **100**, 12 590-12 595. (10.1073/pnas.1735443100) PMC240662 14583590

[6] Benesová M, Schäfer M, Bauder-Wüst U, Afshar-Oromieh A, Kratochwil C, Mier W, Haberkorn U, Kopka K, Eder M. 2015 Preclinical evaluation of a tailor-made DOTA-conjugated PSMA inhibitor with optimized linker moiety for imaging and endoradiotherapy of prostate cancer. J. Nucl. Med. **56**, 914-920. (10.2967/jnumed.114.147413) 25883127

[7] Kratochwil C et al. 2016 PSMA-targeted radionuclide therapy of metastatic castration-resistant prostate cancer with ^177^Lu-labeled PSMA-617. J. Nucl. Med. **57**, 1170-1176. (10.2967/jnumed.115.171397) 26985056

[8] Hofman MS et al. 2018 [^177^Lu]-PSMA-617 radionuclide treatment in patients with metastatic castration-resistant prostate cancer (LuPSMA trial): a single-centre, single-arm, phase 2 study. Lancet Oncol. **19**, 825-833. (10.1016/S1470-2045(18)30198-0) 29752180

[9] Kratochwil C, Bruchertseifer F, Rathke H, Hohenfellner M, Giesel FL, Haberkorn U, Morgenstern A. 2018 Targeted α-therapy of metastatic castration-resistant prostate cancer with ^225^Ac-PSMA-617: swimmer-plot analysis suggests efficacy regarding duration of tumor control. J. Nucl. Med. **59**, 795-802. (10.2967/jnumed.117.203539) 29326358

[10] Sartor O et al. 2021 Lutetium-177-PSMA-617 for metastatic castration-resistant prostate cancer. N. Engl. J. Med. **385**, 1091-1103. (10.1056/NEJMoa2107322) 34161051 PMC8446332

[11] Sjögreen Gleisner K et al. 2022 EANM dosimetry committee recommendations for dosimetry of 177Lu-labelled somatostatin-receptor- and PSMA-targeting ligands. Eur. J. Nucl. Med. Mol. Imaging **49**, 1778-1809. (10.1007/s00259-022-05727-7) 35284969 PMC9015994

[12] Seifert R, Kessel K, Schlack K, Weckesser M, Bögemann M, Rahbar K. 2020 Radioligand therapy using [177Lu]Lu-PSMA-617 in mCRPC: a pre-VISION single-center analysis. Eur. J. Nucl. Med. Mol. Imaging **47**, 2106-2112. (10.1007/s00259-020-04703-3) 32062682 PMC7338828

[13] Kuo HT, Merkens H, Zhang Z, Uribe CF, Lau J, Zhang C, Colpo N, Lin KS, Bénard F. 2018 Enhancing treatment efficacy of ^177^Lu-PSMA-617 with the conjugation of an albumin-binding motif: preclinical dosimetry and endoradiotherapy studies. Mol. Pharm. **15**, 5183-5191. (10.1021/acs.molpharmaceut.8b00720) 30251544

[14] Benešová M, Umbricht CA, Schibli R, Müller C. 2018 Albumin-binding PSMA ligands: optimization of the tissue distribution profile. Mol. Pharm. **15**, 934-946. (10.1021/acs.molpharmaceut.7b00877) 29400475

[15] Wang Z, Jacobson O, Tian R, Mease RC, Kiesewetter DO, Niu G, Pomper MG, Chen X. 2018 Radioligand therapy of prostate cancer with a long-lasting prostate-specific membrane antigen targeting agent ^90^Y-DOTA-EB-MCG. Bioconjug. Chem. **29**, 2309-2315. (10.1021/acs.bioconjchem.8b00292) 29865797 PMC6444910

[16] Umbricht CA, Benešová M, Schibli R, Müller C. 2018 Preclinical development of novel PSMA-targeting radioligands: modulation of albumin-binding properties to improve prostate cancer therapy. Mol. Pharm. **15**, 2297-2306. (10.1021/acs.molpharmaceut.8b00152) 29684274

[17] Arano Y, Mukai T, Uezono T, Wakisaka K, Motonari H, Akizawa H, Taoka Y, Yokoyama A. 1994 A biological method to evaluate bifunctional chelating agents to label antibodies with metallic radionuclides. J. Nucl. Med. **35**, 890-898. 8176478

[18] Uehara T, Yokoyama M, Suzuki H, Hanaoka H, Arano Y. 2018 A gallium-67/68–labeled antibody fragment for immuno-spect/pet shows low renal radioactivity without loss of tumor uptake. Clin. Cancer Res. **24**, 3309-3316. (10.1158/1078-0432.CCR-18-0123) 29666303

[19] Zhang M et al. 2019 Improving the theranostic potential of Exendin 4 by reducing the renal radioactivity through brush border membrane enzyme-mediated degradation. Bioconjug. Chem. **30**, 1745-1753. (10.1021/acs.bioconjchem.9b00280) 31181890

[20] Bendre S, Zhang Z, Kuo HT, Rousseau J, Zhang C, Merkens H, Roxin Á, Bénard F, Lin KS. 2020 Evaluation of Met-Val-Lys as a renal brush border enzyme-cleavable linker to reduce kidney uptake of 68Ga-labeled DOTA-conjugated peptides and peptidomimetics. Molecules **25**, 1-21. (10.3390/molecules25173854) PMC7503470 32854201

[21] Cleeren F, Tshibangu T, Deroose C, Bormans G. 2019 Evaluation of potential cleavable linkers for reduction of renal accumulation of radiolabeled peptides and antibody fragments using mouse renal brush border membranes. J. Labelled Comp. Radiopharm. **62**, S555-S556.

[22] Breeman W, de Zanger R, Chan H, Blois E. 2015 Alternative method to determine specific activity of ^177^Lu by HPLC. Curr. Radiopharm. **8**, 119-122. (10.2174/1874471008666150312162340) 25771376

[23] Breeman WAP, Chan HS, de Blois E. 2014 Determination of peptide content and purity of DOTA-peptides by metal ion titration and UPLC: an alternative method to monitor quality of DOTA-peptides. J. Radioanal. Nucl. Chem. **302**, 825-830. (10.1007/s10967-014-3248-1)

[24] Deberle LM, Benešová M, Umbricht CA, Borgna F, Büchler M, Zhernosekov K, Schibli R, Müller C. 2020 Development of a new class of PSMA radioligands comprising ibuprofen as an albumin-binding entity. Theranostics **10**, 1678-1693. (10.7150/thno.40482) 32042329 PMC6993238

[25] Dumelin CE et al. 2008 A portable albumin binder from a DNA-encoded chemical library. Angew. Chemie – Int. Ed. **47**, 3196-3201. (10.1002/anie.200704936) 18366035

[26] Müller C, Struthers H, Winiger C, Zhernosekov K, Schibli R. 2013 DOTA conjugate with an albumin-binding entity enables the first folic acid–targeted ^177^Lu-radionuclide tumor therapy in mice. J. Nucl. Med. **54**, 124-131. (10.2967/jnumed.112.107235) 23236020

[27] Kuo HT, Lin KS, Zhang Z, Uribe CF, Merkens H, Zhang C, Benard F. 2021 ^177^Lu-labeled albumin-binder–conjugated PSMA-targeting agents with extremely high tumor uptake and enhanced tumor-to-kidney absorbed dose ratio. J. Nucl. Med. **62**, 521-527. (10.2967/jnumed.120.250738) 32859704 PMC8049373

[28] Melis M, Vegt E, Konijnenberg MW, De Visser M, Bijster M, Vermeij M, Krenning EP, Boerman OC, De Jong M. 2010 Nephrotoxicity in mice after repeated imaging using ^111^In-labeled peptides. J. Nucl. Med. **51**, 973-977. (10.2967/jnumed.109.074310) 20484435

[29] Suzuki C, Uehara T, Kanazawa N, Wada S, Suzuki H, Arano Y. 2018 Preferential cleavage of a tripeptide linkage by enzymes on renal brush border membrane to reduce renal radioactivity levels of radiolabeled antibody fragments. J. Med. Chem. **61**, 5257-5268. (10.1021/acs.jmedchem.8b00198) 29869881

[30] Uehara T, Kanazawa N, Suzuki C, Mizuno Y, Suzuki H, Hanaoka H, Arano Y. 2020 Renal handling of ^99m^Tc-labeled antibody fab fragments with a linkage cleavable by enzymes on brush border membrane. Bioconjug. Chem. **31**, 2618-2627. (10.1021/acs.bioconjchem.0c00541) 33085454

[31] Bouvet V, Wuest M, Jans HS, Janzen N, Genady AR, Valliant JF, Benard F, Wuest F. 2016 Automated synthesis of [^18^F]DCFPyL via direct radiofluorination and validation in preclinical prostate cancer models. EJNMMI Res. **6**, 1-15. (10.1186/s13550-016-0195-6) 27142881 PMC4854855

[32] De León-Rodríguez LM, Kovacs Z. 2008 The synthesis and chelation chemistry of DOTA–peptide conjugates. Bioconjug. Chem. **19**, 391-402. (10.1021/bc700328s) 18072717

[33] Liu S, Ellars CE, Edwards DS. 2003 Ascorbic acid: useful as a buffer agent and radiolytic stabilizer for metalloradiopharmaceuticals. Bioconjug. Chem. **14**, 1052-1056. (10.1021/bc034109i) 13129412

[34] Liu S, Edwards DS. 2001 Stabilization of ^90^Y-labeled DOTA-biomolecule conjugates using gentisic acid and ascorbic acid. Bioconjug. Chem. **12**, 554-558. (10.1021/bc000145v) 11459460

[35] de Blois E, Sze Chan H, Konijnenberg M, de Zanger R, Breeman WAP. 2013 Effectiveness of quenchers to reduce radiolysis of 111In- or 177Lu-labelled methionine-containing regulatory peptides. Maintaining radiochemical purity as measured by HPLC. Curr. Top. Med. Chem. **12**, 2677-2685. (10.2174/1568026611212230005) 23339763

[36] de Zanger RMS, Chan HS, Breeman WAP, de Blois E. 2019 Maintaining radiochemical purity of [^177^Lu]Lu-DOTA-PSMA-617 for PRRT by reducing radiolysis. J. Radioanal. Nucl. Chem. **321**, 285-291. (10.1007/s10967-019-06573-y)

[37] Hernandez R et al. 2017 Preventing radiobleaching of cyanine fluorophores enhances stability of nuclear/NIRF multimodality imaging agents. Theranostics **7**, 1-8. (10.7150/thno.15124) 28042311 PMC5196880

[38] de Blois E, Sze Chan H, Breeman WAP. 2013 Iodination and stability of somatostatin analogues: comparison of iodination techniques: a practical overview. Curr. Top. Med. Chem. **12**, 2668-2676. (10.2174/1568026611212230004) 23339762

[39] Breeman WAP et al. 2008 Optimised labeling, preclinical and initial clinical aspects of CCK-2 receptor-targeting with 3 radiolabeled peptides. Nucl. Med. Biol. **35**, 839-849. (10.1016/j.nucmedbio.2008.09.006) 19026945

[40] Hindorf C, Glatting G, Chiesa C, Lindén O, Flux G. 2010 EANM Dosimetry Committee guidelines for bone marrow and whole-body dosimetry. Eur. J. Nucl. Med. Mol. Imaging **37**, 1238-1250. (10.1007/s00259-010-1422-4) 20411259

[41] Murce E, de Blois E, van den Berg S, de Jong M, Seimbille Y. 2023 Data from: Synthesis and radiolabeling of PSMA-targeted derivatives containing GYK/MVK cleavable linkers. *Figshare*. (10.6084/m9.figshare.c.6442433) PMC9993039 36908985

